# Impact of the COVID‐19 Pandemic on Cochlear Implant Usage in Children

**DOI:** 10.1002/ohn.70068

**Published:** 2025-11-18

**Authors:** Peter Kfoury, Kathryn Tonkovich, Eun Kyung Jeon, Jordan C. Stout, Hannah Christensen, Megna D. Reddy, Matthew A. Firpo, Albert H. Park

**Affiliations:** ^1^ Department of Otolaryngology–Head and Neck Surgery University of Utah School of Medicine Salt Lake City Utah USA; ^2^ Department of Audiology Primary Children's Hospital–Intermountain Health Care Salt Lake City Utah USA; ^3^ Department of Communication Sciences and Disorders University of Iowa Iowa City Iowa USA; ^4^ Noorda College of Osteopathic Medicine Provo Utah USA; ^5^ Department of Surgery University of Utah School of Medicine Salt Lake City Utah USA; ^6^ Department of Otolaryngology–Head and Neck Surgery and Department of Pediatrics University of Utah School of Medicine Salt Lake City Utah USA

**Keywords:** aural rehabilitation, cochlear implant, COVID‐19, datalogging, hearing hour percentage, pandemic preparedness, pediatric hearing loss

## Abstract

**Objective:**

To evaluate the impact of the COVID‐19 pandemic on cochlear implant (CI) usage in children by comparing hearing hour percentage (HHP) during the pandemic to prepandemic levels.

**Study Design:**

Retrospective Cohort Study.

**Setting:**

Primary Children's Hospital, Utah.

**Methods:**

A retrospective chart review was conducted for children aged 9 months to 18.9 years old who underwent CI between January 2018 and September 2023. Inclusion criteria comprised first‐time CI recipients with at least 1 year of follow‐up data. HHP was calculated by dividing total wear time by the expected daily awake time based on the patient's age. Multivariate linear regression analysis was performed to control for confounding variables of HHP, with demographic, clinical, and procedural variables entered as independent variables.

**Results:**

A total of 156 patients were included. A significant decline in HHP was observed during the pandemic, from 55.0% (SE = 4.3, n = 30) in 2019 to 26.2% (SE = 5.5, n = 22) in 2020. Regression analyses also revealed a significant decrease in HHP during both the early pandemic period (2020‐2021) (*P* = .0014) and late in the pandemic period (2022‐2023) (*P* = .011) compared to pre‐pandemic levels. After controlling for confounders, the late pandemic period remained significantly associated with a reduction in HHP (*β* = −15.6, *P* = .018), but the decline in HHP during the early phase of the pandemic was no longer significant.

**Conclusion:**

Pandemic‐related disruptions led to a decline in pediatric CI use, underscoring the need for targeted support during public health crises.

Cochlear implantation (CI) has become a standard of care for children with severe or profound sensorineural hearing loss (SNHL).^1^, ^2^ The effectiveness of CIs is strongly influenced by the duration of device use.^3^, ^4^ Research has consistently demonstrated that increased daily CI usage correlates with better speech and vocabulary outcomes.^3^, ^4^, ^5^, ^6^ Key factors that have been shown to influence the consistency of CI usage include age at implantation,^3^ longer CI experience,^7^, ^8^ pre‐CI hearing experience,^8^ oral‐aural communication,^9^ and maternal education.^10^

The COVID‐19 pandemic introduced several challenges for children with CI, disrupting follow‐up visits,^11^ device programming sessions,^12^ and rehabilitation,^12^ while also contributing to delays in hearing loss diagnosis nationwide.^13^ In Utah, services for hearing loss detection and diagnosis remained operational throughout the pandemic.^14^ However, pandemic‐related restrictions, such as social distancing, lockdowns, and school closures, reduced children's exposure to verbal communication.^15^

These restrictions have negatively impacted CI utilization among older adults.^16^ Marinelli et al demonstrated a 15% decline in CI utilization among Medicare‐aged patients and nearly 25% among those aged 80 and older in 2020.^16^ It is believed this decline, coupled with missed programming appointments and reduced exposure to complex listening environments, contributed to poorer speech outcomes in elderly CI recipients.^16^, ^17^

There remains a lack of data on how the pandemic influenced CI usage in children. While Marinelli et al found that children—particularly those under 3 years old—experienced minimal disruption, their study only included data through 2020, leaving the impact of later periods unclear.^16^

This study examines pediatric CI usage during the COVID‐19 pandemic using objective datalogging measures. We aimed to determine whether device usage declined during the pandemic compared to prepandemic levels, with the hypothesis that pandemic‐related disruptions led to reduced daily wear time during the first year postimplantation.

## Methods

### Study Design

This retrospective cohort study was approved by the institutional review board (IRB_00146167) of the University of Utah and Primary Children's Hospital (PCH), Utah. We performed a retrospective chart review of patients who underwent CI between January 1, 2018, and September 1, 2023 at Primary Children's Hospital (PCH), Utah. Inclusion criteria comprised patients receiving their first CI, with at least 1 year of follow‐up data available. Patients who had undergone revision surgeries or lacked datalogging information were excluded.

### Independent Variables

We collected various demographic and clinical data that may influence hearing hour percentage (HHP). Independent variables include age at implantation, race/ethnicity, etiology of hearing loss, insurance type, surgeon, type of hearing loss (bilateral symmetric, bilateral asymmetric, or single‐sided deafness), and date of implantation. Age was categorized into 5 groups: infants (less than 12 months old), toddlers (12‐35 months), preschoolers (36‐71 months), elementary‐aged children (6‐12 years), and teenagers (13‐18 years).

### Dependent Variables

Device usage data were obtained from datalog files extracted using manufacturer‐specific programming software (Cochlear Americas, Advanced Bionics, and MED‐EL) during routine audiology visits within the first year of implantation. The total number of hours of device use during the first year was calculated by averaging the wear time for each patient based on all visits within the year. This methodology was chosen because it provides a more comprehensive reflection of device use across the first year. It ensures that wear time estimates are not overly influenced by isolated events, such as device replacements or repairs, and captures a broader picture of usage patterns over time.

For bilateral users, we calculated the average wear time for both ears rather than using the higher wear time from 1 ear, which has been reported in other studies.^3^, ^4^ We opted for this approach because it better reflects the overall device use between routine programming appointments and avoids overestimating wear time. Moreover, we found that the difference between the average and higher wear times was minimal, as most bilateral users tend to use their two devices equally. The mean difference between the average and higher wear times for all bilateral users was 0.41 h/day, with 59 out of 70 (84%) users showing a difference of less than half an hour per day.

### HHP

To assess the difference in wear time relative to a child's waking hours, we calculated the HHP. This value was calculated by determining the expected waking hours for each child based on their age, using the inversion of the median sleep recommendations from the American Academy of Sleep Medicine,^18^ following the methodology described by Gagnon et al.^4^ The equation used to compute HHP was: (total wear time/total awake time) × 100. Table 1 presents the median awake time per day by age group used to calculate the HHP in this study.

**Table 1:** Median Awake Time Per Day by Age Based on Pediatric Sleep Recommendations from the American Academy of Sleep Medicine

Age (months)	Median awake time (h/day)
4‐12	10
13‐35	11.5
36‐71	12.5
72‐155	13.5
156‐228	15

### Audiological Testing

All audiological testing was performed as part of routine CI programming visits by a qualified pediatric audiologist and included auditory brainstem response (ABR) or behavioral testing prior to CI. ABR thresholds >25 dB and behavioral thresholds >20 dB were considered indicative of hearing loss. Hearing loss was categorized by severity based on the pure‐tone average (PTA) of these frequencies: 0.5, 1, 2, and 4 kHz. Severity was defined as mild for 30 to 45 dB (ABR) or 21 to 45 (behavioral), and for both testing methods, moderate for 46 to 70 dB, severe for 71 to 90 dB and profound for >90 dB.^19^ Any hearing loss that exceeded machine limitations was considered profound. Hearing loss was also categorized by laterality. Asymmetric hearing loss was defined as a difference of 10 dB or greater in two consecutive frequencies or 15 dB in any one frequency between ears, and for this study, included instances of unilateral hearing loss.^20^ Single‐sided deafness (SSD) was defined as normal hearing in 1 ear and profound or severe hearing loss in the other ear.

### Statistical Analysis

Descriptive statistics were calculated to summarize the demographic and clinical characteristics of the study cohort, including means and standard errors (SE) for continuous variables, and frequencies and percentages for categorical variables. Chi‐square and Fisher's exact test were performed to detect any demographic differences between the different study groups. Comparisons of continuous variables between groups were conducted using unpaired *t*‐tests or one‐way analysis of variance, as appropriate.

Univariate linear regression analysis was performed to assess the effects of the pandemic on HHP. Multivariate linear regression analysis was performed to address confounders, with demographic, clinical, and procedural variables used as independent variables. Variables included in the regression model were selected based on clinical relevance and univariate analysis significance (*P* < .05). Effects sizes (*β* coefficients), SE, and *P*‐values were reported for each predictor.

All tests were 2‐tailed and a *P* < .05 was considered statistically significant. Analyses were conducted using R (version 4.1.2).

## Results

**Figure 1:**
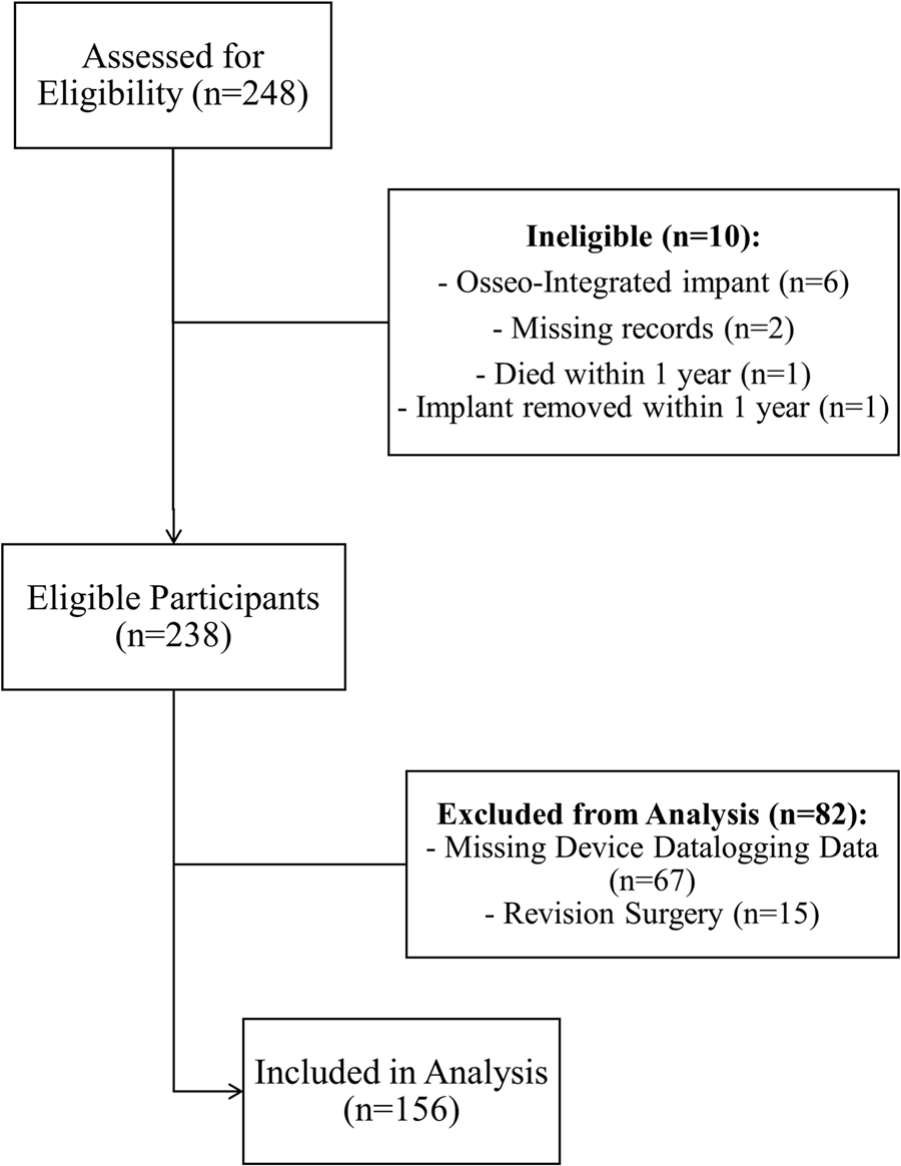
Flowchart illustrating patients included and excluded from the study analysis. Between January 2017 and September 2023, 248 participants were evaluated for eligibility. Of these, 156 participants were included in the final analysis.

A total of 248 participants were screened for eligibility. Ten were excluded due to: osseo‐integrated implant surgery (n = 6), incomplete or missing records (n = 2), postoperative death within 1 year (n = 1), or implant removal due to cerebrospinal fluid leakage (n = 1), resulting in 238 eligible participants. This resulted in 238 participants eligible for inclusion. Subsequently, 82 of the eligible participants were further excluded from the analysis: 67 lacked datalogging information and 15 had revision CI surgery. A total of 156 participants were included in the final analysis (Figure 1).

**Figure 2:**
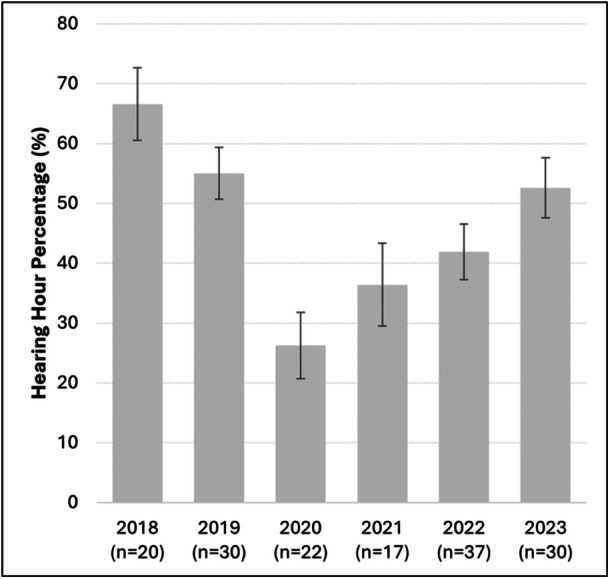
Average yearly hearing hour percentage (HHP) across study period. The error bars represent the standard error of the mean. The sample size for each year, n, is shown below the corresponding bar.

Average HHP varied across the study period with a distinct decline during 2020, as shown in Figure 2, from 66.6% (SE = 6.1) in 2018 and 55.0% (SE = 4.3) in 2019 to 26.2% (SE = 5.5) in 2020. In the following years, a gradual recovery was observed, with HHP increasing to 41.9% (SE = 4.7) in 2022 and 52.6% (SE = 5.0) in 2023. Based on these trends, study participants were grouped into 3 time periods: pre‐COVID (2018‐2019), early pandemic (2020‐2021), and late pandemic (2022‐2023).

The demographic and clinical characteristics of the study cohorts (n = 156) are summarized in Table 2. Insurance coverage differed significantly across study groups (Fisher's Exact test, *P* < .001), with 64% of participants in the early pandemic group (n = 25) having public insurance, compared to 33% in the overall cohort. Surgical care was provided by 4 surgeons, with notable variation in case distribution across time (Fisher's Exact Test, *P* < .001). Surgeon I retired during the early pandemic phase, while surgeons III and IV started performing those surgeries during the pandemic. Surgeon II performed procedures throughout the study period. There were no significant differences between the 3 cohorts in terms of age, hearing loss profile, surgery performed (bilateral vs unilateral CI), CI manufacturer, or etiology of hearing loss.

**Table 2:** Demographic and Clinical Characteristics of Study Participants (n = 156) Stratified by Period of Implantation

Feature	Pre‐COVID	Early pandemic	Late pandemic	n (%)	*P*‐value
Gender					
Male	28	19	32	79 (51%)	.65
Female	22	20	35	77 (49%)	
Race/ethnicity					
White, non‐Hispanic	34	23	48	105 (67%)	.21
White, Hispanic	11	9	8	28 (18%)	
Unknown	1	2	7	10 (6%)	
Asian	2	1	3	6 (4%)	
Other	2	4	1	7 (4%)	
Insurance					
Medicaid	9	25	17	51 (33%)	<.001
Private	41	14	50	105 (67%)	
Surgeon					
I	25	5	0	30 (19%)	<.001
II	25	17	13	55 (35%)	
III	0	17	29	46 (29%)	
IV	0	0	25	25 (16%)	
HL type					
Symmetric	33	20	39	92 (59%)	.29
SSD	11	7	18	36 (23%)	
Asymmetric	6	11	10	27 (17%)	
Manufacturer					
Cochlear Americas	38	29	52	119 (76%)	.27
Advanced Bionics	4	4	11	19 (12%)	
Med El	8	6	4	18 (12%)	
Surgery					
Right CI	13	11	26	50 (32%)	.69
Left CI	13	9	14	36 (23%)	
Bilateral simultaneous CI	20	18	23	61 (39%)	
Bilateral sequential CI	4	1	4	9 (6%)	
Age category					
Infant (less than 12 mo)	1	2	5	8 (5%)	.62
Toddler (13‐35 mo)	14	14	23	51 (33%)	
Preschooler (36‐71 mo)	11	10	10	31 (20%)	
Elementary (72‐155 mo)	14	6	19	39 (25%)	
Teenage (156‐228 mo)	10	7	10	27 (17%)	
Etiology					
Idiopathic	12	11	28	51 (33%)	.30
Anatomical Defects	11	5	10	26 (17%)	
cCMV	11	6	8	25 (16%)	
ANSD	8	5	7	20 (13%)	
Genetic	2	7	9	18 (12%)	
Other	6	5	5	16 (10%)	

Statistical significance was assessed through Chi‐square tests, Fisher's Exact Test, where appropriate.

Abbreviations: CI, cochlear implant; HL, hearing loss; mo, months old; n, sample size; SSD, single‐sided deafness.

The mean HHP for the entire cohort was 49.3% (SE = 2.2). The age at implantation averaged 6.5 years (SE = 0.4). At the time of surgery, 33% of participants were toddlers aged from 13 to 35 months old, 20% were preschoolers between 3 and 5.9 years old, 25% were elementary school‐aged between 6 and 12.9 years old, and 17% were teenagers between 13 and 18.9 years old. Only 8 individuals (5%) were implanted before the age of 1. Less than a quarter of patients (n = 27, 17%) reported full‐time usage of their devices, defined as at least 80% of HHP. Within our cohort, 57 (36.5%) patients wore their device for at least 8 hours per day within the first year following implantation. The analysis of HHP across various demographic and clinical factors is detailed in Table 3.

**Table 3:** Hearing Hour Percentage (HHP) by Demographics, Clinical Characteristics, and Surgical Variables

	Average HHP	SE	n	*P*‐value
Gender				
Male	47.88	3.29	79	.498
Female	50.92	3.03	77	
Age category				
Infant	50.99	10.72	8	<.0001
Toddler	33.35	3.41	51	
Preschooler	52.32	5.13	31	
Elementary	62.93	3.73	39	
Teenage	56.23	5.07	27	
Race/ethnicity				
White, non‐Hispanic	53.48	2.63	105	.119
White, Hispanic	41.69	5.29	28	
Unknown	39.47	8.60	10	
Asian	45.35	15.35	6	
Other	36.22	10.79	7	
Insurance				
Public	35.83	3.71	51	<.0001
Private	55.96	2.57	105	
HL type				
Bilateral symmetric	51.42	2.98	92	.297
SSD	42.89	4.43	36	
Bilateral asymmetric	50.02	5.25	27	
Surgery				
Right CI	48.60	3.92	50	.0005
Left CI	55.08	4.35	36	
Bilateral simultaneous CI	42.05	3.48	61	
Bilateral sequential CI	80.65	4.94	9	
Surgeon				
I	52.78	4.43	30	.82
II	50.32	3.78	55	
III	47.22	4.45	46	
IV	47.21	5.76	25	
Manufacturer				
Cochlear	47.50	2.61	119	.126
AB	61.53	6.00	19	
MedEL	49.00	5.73	18	
Period				
Pre‐COVID	59.65	3.50	50	.0035
Early pandemic	40.84	4.33	39	
Late pandemic	46.69	3.46	67	
Etiology				
Idiopathic	51.62	3.91	51	.235
Anatomical defects	58.79	5.39	26	
cCMV	41.41	5.09	25	
ANSD	43.28	4.45	20	
Genetic	51.46	7.6	18	
Other	44.69	8.46	16	

Abbreviations: AB, advanced bionics; ANOVA, analysis of variance; ANSD, auditory neuropathy spectrum disorder; cCMV, congenital cytomegalovirus; CI, cochlear implant; HHP, hearing hour percentage; n, sample size; SE, standard error; SSD, single‐sided deafness.

**Figure 3:**
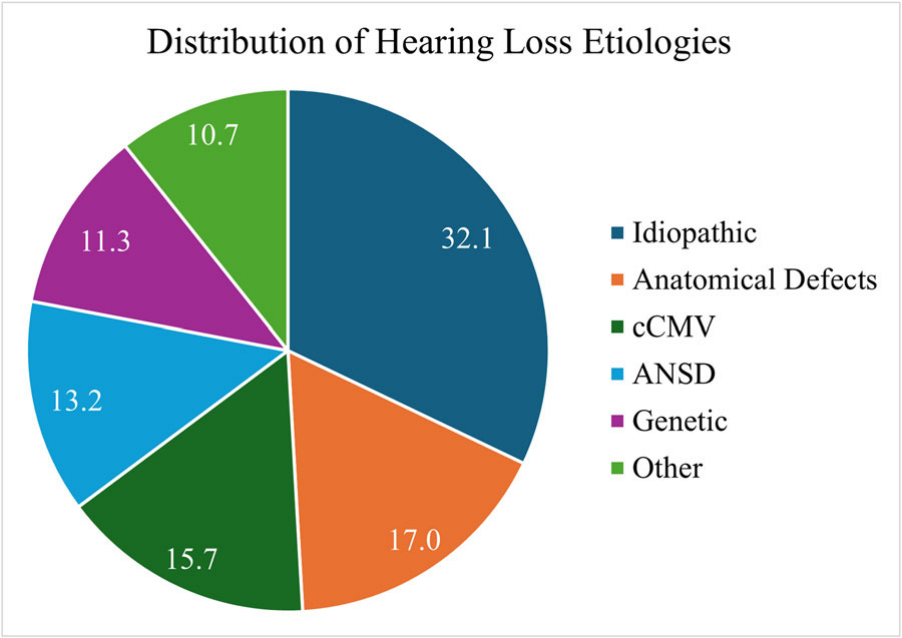
Etiologies of hearing loss in patients undergoing cochlear implantation. Abbreviations: ANSD, auditory neuropathy spectrum disorder; cCMV, congenital cytomegalovirus.

The distribution of etiologies for hearing loss for our cohort is shown in Figure 3. The most common cause of hearing loss was idiopathic, accounting for 32.1% of cases, followed by anatomical defects (17.0%)—for example, enlarged vestibular aqueduct. The third most common cause was congenital cytomegalovirus (cCMV), accounting for 15.7% of cases. The etiologies classified as “Other” included: bacterial meningitis (n = 7, 4.4%), exposure to ototoxic drugs (n = 4, 2.5%), traumatic injury (n = 2, 1.3%), congenital syphilis (n = 1, 0.6%), maternal drug use (n = 1, 0.6%), lightning strike (n = 1, 0.6%), and labyrinthitis (n = 1, 0.6%).

A univariate linear regression model, with the pre‐COVID period as reference category, demonstrated a significant decline in HHP during the early pandemic period with an estimated decrease of 18.8% compared to the pre‐COVID period (*P* = .0014) (Table 4). Although partial recovery was observed in the late pandemic period, HHP remained significantly lower than pre‐COVID levels, with an estimated reduction of 13.0%.

**Table 4:** Linear Regression Analysis Evaluating the Impact of the Pandemic Onset on Hearing Hours Percentage

		Estimate	Std. Error	*t*‐value	*P*‐value
(Intercept)		59.66	3.83	15.56	<.0001
Period	Early Pandemic	−18.82	5.79	−3.25	.0014
	Late Pandemic	−12.97	5.07	−2.56	.0114

Implantation before the onset of the pandemic was considered the reference variable. Residual standard error was 27.11 on 153 degrees of freedom (*F* = 5.9, *P* = .0035). Multiple R‐squared: 0.071, Adjusted R‐squared: 0.059.

Multivariate linear regression analysis controlled for known clinical confounders, including age category, hearing loss profile (bilateral symmetric, bilateral asymmetric, and SSD), type of procedure, surgeon, and insurance (Table 5). After controlling for these variables, the late pandemic period remained significantly associated with a reduction in HHP (*β* = −15.6, *P* = .018), whereas the early pandemic period showed a nonsignificant decline (*β* = −11.2, *P* = .075). One possible explanation is that a disproportionately large representation of Medicaid‐insured patients underwent CI during the early pandemic period (Table 2; *P* < .001). Since Medicaid insurance itself was independently associated with lower HHP (*β* = −15.2, *P* = .0012), this overrepresentation may have overshadowed the impact of the early pandemic period in the multivariate regression analysis, making the decline appear non‐significant.

**Table 5:** Linear Regression Analysis of Factors Influencing Hearing Hours Percentage

	Estimate	Std. Error	*t*‐value	*P*‐value
(Intercept)				
Period	30.04	6.08	4.94	<.0001
Early pandemic	−11.18	6.23	−1.79	.075
Late pandemic	−15.59	6.52	−2.39	.018
Age				
Infant	15.45	9.03	1.71	.089
Preschooler	19.00	5.32	3.58	.00048
Elementary	25.33	5.29	4.79	<.0001
Teenage	14.59	6.43	2.27	.025
Type of HL				
SSD	−20.31	5.67	−3.58	.00047
Bilateral asymmetric	−7.64	5.76	−3.58	.187
Insurance				
Private	15.19	4.59	3.31	.0012
Surgeon				
II	2.32	5.78	0.40	.689
III	8.52	7.43	1.15	.254
IV	9.64	8.76	1.099	.273
Procedure				
Right CI	5.72	5.99	0.96	.341
Left	11.35	6.56	1.73	.086
Bilateral sequential CI	30.51	8.53	3.58	.00048

The reference variables included toddler age group, pre‐COVID period, bilateral symmetric hearing loss, public insurance, surgeon I, and bilateral simultaneous cochlear implantation. The model explained 41% of the variability in HHP (*R* ^2^ = 0.41) and 34% after adjusting for model complexity (Adjusted *R* ^2^ = 0.34). The overall model was statistically significant (*F* (15, 139) = 6.32, *P* < .0001).

Abbreviations: CI, cochlear implant; HL, hearing loss; SSD, single‐sided deafness.

Children with SSD were also significantly less likely to wear their CI compared to children with bilateral symmetric HL (*β* = −20.3, *P* = .00047). Age was also a significant predictor of HHP, with preschoolers (*β* = 19.0, *P* = .00048), elementary‐aged children (*β* = 25.3, *P* < .0001), and teenagers (*β* = 14.6, *P* = .025) demonstrating higher HHP compared to toddlers. After stratifying study periods by age groups, the findings revealed that children implanted between 3 and 12.9 years old were the most affected by the pandemic, exhibiting noticeable declines in HHP. HHP dropped for preschoolers from 70.0% (SE = 7.0) pre‐COVID to 38.1% (SE = 8.2) in the early pandemic phase, as well as for elementary‐aged children dropping from 71.7% (SE = 4.8) pre‐COVID to 52.6% (SE = 10.0) during the early phases of the pandemic.

## Discussion

The primary aim of this study was to investigate the impact of the COVID‐19 pandemic on CI usage in the pediatric population during the first‐year postimplantation. Our findings revealed a significant decline in HHP during the early and late stages of the pandemic. To our knowledge, this is the first study to specifically assess the effects of the pandemic on HHP in the pediatric CI population. These results are consistent with prior research in adult CI users, which also demonstrated a decline of up to 25% in device usage among adults over 80 years old during the pandemic.^16^

Several factors may have contributed to the observed decline in HHP, such as restricted social gatherings, lockdowns, and school closures. We postulate that these circumstances reduced children's exposure to spoken communication.^15^, ^17^ As a result, pediatric CI users—particularly those between 3 and 13 years of age—experienced a notable reduction in daily device wear time. The mean age at implantation (6.5 years), average daily wear time (6.4 hours), and mean HHP (49.3%) are comparable to values reported in similar studies.^21^, ^22^, ^23^ Gagnon et al. demonstrated an average HHP of 63% in children implanted between 2013 and 2017,^4^ which aligns with our pre‐COVID average of 60%. The percentage of children achieving full‐time device use (17%) is notably lower than in prior research, likely due to the high proportion of children implanted during the pandemic. Indeed, Park et al reported that 43% of children implanted reached full‐time usage within a year following implantation.^3^

Age at implantation, insurance coverage, SSD, and bilateral sequential CI surgeries were key predictors of CI usage. Our linear regression model accounted for 40% of the variability in CI usage. Toddlers aged 1 to 3 years had an HHP that was, on average, 20% lower than that of children between 3 and 13 years old, after controlling for other variables. This aligns with previous findings that younger children take longer to reach full‐time use.^10^, ^23^ One possible explanation is that children implanted at an older age have a longer pre‐CI hearing experience, which is associated with higher CI usage and better speech outcomes.^7^, ^8^ Given this, early implantation—between 6 and 12 months—is strongly recommended to optimize spoken language development.^24^

Children with SSD also exhibited an HHP approximately 20% lower than those with bilateral symmetric hearing loss, even when controlling for other predictors. Macielak et al. reported similar findings in a cohort of 66 SSD patients, where only 10 of 54 (19%) were consistent CI users (datalogging ≥6 hours per day).^25^ Polonenko et al, in a prepandemic cohort, reported a mean CI use of 7.4 h/day (range ~3.5–11.2 hours/day) among SSD children.^26^ In that study, older SSD children used their CI more consistently than younger ones, and CI use was maintained across various listening environments, especially in speech‐in‐noise settings.^26^ Importantly, consistent CI use in Polonenko's cohort was likely supported by several factors, including shorter duration of unilateral deprivation, strong parental motivation (as all cases involved off‐label implantation), and the early adoption of structured routines postactivation.^26^ In contrast, our findings suggest that in routine clinical practice—particularly during the pandemic—many SSD children faced additional barriers to consistent CI use, possibly due to pandemic‐related disruptions in follow‐up care, counseling, and school‐based support.

Socioeconomic disparities were evident as well. Children with private insurance had an HHP approximately 10% higher than those with public insurance (i.e., Medicaid), consistent with findings from previous studies.^10^ Wiseman and Warner‐Czyz similarly reported that children with Medicaid coverage exhibited lower average CI use (7.6 h/day, range 0.1‐15.5), with reduced wear time associated with younger age, additional disabilities, lower maternal education, and smaller dynamic ranges.^10^ Awad et al. showed that privately insured children used their CI 1.2 hours more per day than publicly insured peers, and that even small increases in daily wear time correlated with significant improvements in speech perception (2.6% gain per additional hour).^27^ Their study also found that lower socioeconomic status was linked to reduced follow‐up care, as children from lower‐income families were less likely to attend later datalogging visits. Our findings add to this growing body of evidence, suggesting that pandemic‐related disruptions may have further exacerbated socioeconomic‐related disparities in CI use. The combined effects of public insurance status and pandemic limitations likely introduced additional barriers to consistent device use in this population. These results underscore the importance of targeted interventions—such as enhanced access to follow‐up care, caregiver education, and school‐based supports—to mitigate disparities in CI usage and optimize outcomes for children from lower socioeconomic backgrounds.

### Clinical Implications

These findings underscore the importance of maintaining consistent CI use during the first year postimplantation, even during periods of healthcare disruption. Reduced usage during the COVID‐19 pandemic highlights the need for clinicians to provide proactive counseling to families on the value of daily device wear, and to implement strategies such as remote programming, telehealth‐based habilitation, and enhanced caregiver education. Early identification of patients at risk for reduced device use—such as those with SSD or from lower socioeconomic backgrounds—can help audiologists and implant teams tailor interventions to promote consistent CI use and optimize long‐term outcomes.

### Limitations

A total of 248 participants were evaluated for eligibility, with 156 ultimately included in the study cohort. The exclusion of participants lacking datalogging information (n = 67) may have influenced the study's generalizability, as these individuals may have followed up with non‐PCH audiologists. Nevertheless, the final cohort provides the largest sample of pediatric CI users in a single study evaluating datalogging information.

Another limitation was the inability to account for certain variables that may have affected HHP, such as maternal education. Prior research by Marnane and Ching found that pediatric CI users whose mothers did not have a university education were more likely to have reduced device usage within the first 3 years.^28^

## Conclusion

Pediatric CI usage declined during the COVID‐19 pandemic, particularly among preschoolers and elementary‐aged children. The drop in HHP remained notable after controlling for several other key predictors of CI usage, including age at implantation, type of insurance coverage, single‐sided deafness, and bilateral sequential CI. These findings highlight the importance of targeted interventions—such as enhanced family counseling, school‐based support, and flexible follow‐up care—to sustain consistent CI use during periods of healthcare disruption and beyond. Ongoing efforts are needed to mitigate disparities and optimize outcomes for all pediatric CI recipients.

## Author Contributions

**Peter Kfoury:** data collection, data analysis, visualization, interpretation, drafting, critical revision; **Kathryn Tonkovich:** study design, interpretation, drafting, critical revision; **Eun Kyung Jeon:** study design, interpretation, drafting, critical revision; **Jordan C. Stout:** data collection, critical revision; **Hannah Christensen:** data collection, critical revision; **Megna D. Reddy:** data interpretation, drafting, critical revision; **Matthew A. Firpo:** data interpretation, critical revision; **Albert H. Park:** study design, data interpretation, critical revision, supervision.

## Disclosures

### Competing interests

None.

### Funding source

None.
